# Cartilage matrix changes in contralateral mobile knees in a rabbit model of osteoarthritis induced by immobilization

**DOI:** 10.1186/s12891-015-0679-y

**Published:** 2015-08-25

**Authors:** Qiang Zhou, Bo Wei, Shuai Liu, Fengyong Mao, Xiang Zhang, Jun Hu, Jin Zhou, Qingqiang Yao, Yan Xu, Liming Wang

**Affiliations:** Department of Orthopedics, Nanjing First Hospital, Nanjing Medical University, Nanjing, Jiangsu 210006 China; Cartilage Regeneration Center, Nanjing First Hospital, Nanjing Medical University, Nanjing, Jiangsu 210006 China; Digital Medicine Institute, Nanjing Medical University, Nanjing, Jiangsu 210006 China; Department of Orthopedics, No. 454 Hospital of People’s Liberation Army, Nanjing, Jiangsu China

## Abstract

**Background:**

Many researches have investigated the changes associated with immobilization-induced osteoarthritis (OA). However, there are only few studies focusing on the effect of unilateral knee immobilization on cartilage matrix changes in the contralateral mobile knee. The aim of the present study was to investigate the influence of immobilization on the cartilage matrix in the contralateral mobile knees in a rabbit model of OA induced by immobilization.

**Methods:**

Right knees (experimental knees) of eighteen mature female rabbits were immobilized at an extension of 180° with orthopedic casting tape for 2, 4, or 8 weeks. Left knees (contralateral knees) of the immobilized rabbits were not subjected to immobilization. The knees of six non-immobilized rabbits were designated as control knees. Following immobilization, cartilage specimens from the medial femoral condyle underwent macroscopic, histological, immunohistochemical, and biochemical evaluations.

**Results:**

Roughness of cartilage surface was detected in the experimental knees at 2 weeks, and cartilage degeneration was further developed. In the contralateral knee, cartilage showed degenerative changes after 4 weeks. Safranin-O staining and glycosaminoglycan (GAG) contents were reduced in the experimental knees following immobilization and in the contralateral intact knees after 4 and 8 weeks. Type II collagen staining was gradually reduced, type I collagen accumulation was obviously detected in the upper and middle layers of cartilage in experimental knees after 8 weeks, and the collagen orientation was gradually disorganized in both knees at 4 and 8 weeks. For both experimental and contralateral knees, collagen contents were significantly decreased at 8 weeks, and Mankin and Osteoarthritis Research Society International (OARSI) scores increased over time.

**Conclusion:**

OA developed in the contralateral intact knee with the progress of OA in the immobilized knee in a rabbit model of immobilization-induced OA.

## Background

Osteoarthritis (OA) is a challenging global health problem. In the early-stage of OA, no obvious clinical symptoms and radiographic findings can be detected. With the progression of OA, the patients often suffer from severe pain and progressive destruction of articular cartilage; most of the patients with late-stage OA will inevitably undergo prosthetic arthroplasty [1]. Patients with unilateral knee OA who have undergone primary total knee arthroplasty (TKA) are likely to develop OA in the contralateral knee and have a 37.2 % 10-year risk of TKA in the contralateral knee [2]. Moreover, the risk of contralateral TKA does not show a significant association with either sex, age, BMI, or side of initial TKA [2]. Early postoperative rehabilitation interventions after unilateral TKA including improving the strength of contralateral limbs do not only improve the outcome in the operated knee but also delay the progress of OA in the contralateral knee [3]. Another group reported that patients with unilateral knee OA have a high risk of developing OA in the contralateral knee within 10 years; moreover, reduction in the medial loading in both knees could prevent OA progression in the contralateral knee [4]. Therefore, early detection and intervention have great significance in preventing and treatment of OA [5, 6]. However, cartilage specimens from patients with early OA cannot be easily obtained for further analysis.

To date, many groups have used a variety of animal models to investigate the changes associated with OA [7, 8]. Among them, joint immobilization can safely and conveniently induce OA [9]. Mechanical stress plays a role in the structure and function of articular cartilage, and joint immobilization could influence the biomechanical, morphological, and biochemical properties of cartilage [10]. Glycosaminoglycan (GAG) content in articular cartilage decreased after 11 weeks of immobilization, but the normal concentration of GAGs was restored at most sites after 50 weeks of remobilization in a canine model [11]. Moreover, the mechanical properties of cartilage in both immobilized and contralateral knee joints have been reported to be affected after 4-week immobilization in a canine model [12]. Thus, the characteristics of cartilage in contralateral knees were different from those in knees of control canines which were not subjected to immobilization [12, 13]. It is of great significance to detect the cartilage matrix changes in the contralateral intact knee following immobilization. However, there are currently only few studies focusing on the effect of unilateral knee immobilization on cartilage matrix changes in the contralateral intact knee [13, 14].

Therefore, the goal of the present study was to investigate the influence of immobilization on changes in the cartilage matrix in the contralateral knee in a rabbit model of OA induced by immobilization. We hypothesized that OA also developed in the contralateral mobile knee with the progress of OA in the immobilized knee in the rabbit model of immobilization-induced OA.

## Methods

### Animal model

All experimental procedures were approved by the Animal Research Committee and the Institutional Review Board of Nanjing Medical University. Right knees (experimental knees) of eighteen New Zealand white rabbits (female; 4 months old; weighting 3.2–3.8 kg) were immobilized at 180° of extension with orthopedic casting tape (Nanjing Shuangwei Biotech. Co., Ltd., Jiangsu, China). Left knees (contralateral knees) of the immobilized rabbits were not subjected to immobilization. All immobilized rabbits were permitted to bear weight normally. The tapes were removed after 2, 4, or 8 weeks of immobilization and samples were collected immediately after removing the tapes. The knees of six non-immobilized rabbits were designated as control knees.

### Macroscopic observation and histological assessment

After immobilization, the gross appearance of the femoral condyle was photographed and assessed. The collected osteochondral samples from the intermediate region of the medial femoral condyles were fixed with 10 % neutral formalin for 24 h, decalcified with 5 % nitric acid for 4 days, dehydrated through a series of graded ethanol solutions, cleared in xylene, and embedded in paraffin. Sections, 5 μm in thickness, were stained with Safranin-O/Fast-Green (SO) for GAG distribution and with Sirius Red for collagen anisotropy, respectively. Staining results were evaluated separately using light microscopy (Ci-L; Nikon, Tokyo, Japan) and polarized light microscopy (Ci-L).

### Immunohistochemical evaluation

Separate sections, 5 μm in thickness, were sequentially incubated with proteinase K for 30 min, followed by 3 % hydrogen peroxide/methanol for 10 min, and 10 % goat serum for 30 min. Sections were incubated with a mouse anti-type II collagen antibody (Novus Biologicals, Littleton, CO, USA) and a mouse anti-type I collagen antibody (Sigma, St. Louis, MO, USA) for 90 min, respectively. Then, the sections were incubated with a secondary goat anti-mouse antibody (Nanjing KeyGen Biotech. Co., Ltd., Jiangsu, China) for 30 min, 3,3′-diaminobenzidine tetrahydrochloride (DAB) solution for 5 min, counterstained with hematoxylin for 8 min, and mounted with neutral balsam. Staining results were evaluated using light microscopy (Ci-L).

### Biochemical analysis

Full-thickness cartilage specimens were harvested from the whole medial femoral condyle and the GAG and total collagen contents were separately measured using a Blyscan^TM^ sulfated GAG and a Sircol Collagen Assay Kit (both kits from Biocolor, Newtonabbey, UK) as described previously [15]. For GAG analysis, cartilage samples were digested with papain, mixed with the Blyscan dye reagent, and centrifuged at 12,000 rpm for 10 min. Then, the dissociation reagent was added to the precipitate and the absorbance at 656 nm was determined in a microplate reader (Bio-Rad Laboratories, Richmond, CA). For collagen analysis, cartilage samples were digested with pepsin, mixed with the Sircol dye reagent, and centrifuged at 12,000 rpm for 10 min. The precipitate was washed with acid-salt wash reagent and dissolved in alkali reagent, and then the absorbance at 555 nm was determined in a microplate reader (Bio-Rad Laboratories).

### Histopathological scores

The Mankin and Osteoarthritis Research Society International (OARSI) scoring systems [16, 17] were used to evaluate the progression of OA. The Mankin score was determined in four stained sections from each medial femoral condyle sample by a blinded observer. For OARSI scoring, grade and stage of the medial femoral condyle was evaluated in four sections from each cartilage specimen by a blinded observer. OARSI scores were calculated by multiplying the grade by the stage.

### Statistical analysis

All values were expressed as the mean ± standard deviation (SD). Statistical analyses were performed using SPSS software (Version 13.0, Chicago, IL: SPSS Inc.). One-way analysis of variance and Fisher’s least significant difference *post-hoc* test was used to evaluate the differences in biochemical contents, and in Mankin and OARSI scores of experimental or contralateral knees at 2, 4, and 8 weeks of immobilization. The unpaired Student’s t-test was used to compare the means of GAG and collagen contents, and of Mankin and OARSI scores of experimental *vs*. contralateral knees at specific time points of immobilization. *P* < 0.05 was considered statistically significant.

## Results

### Gross observation

**Fig. 1:**
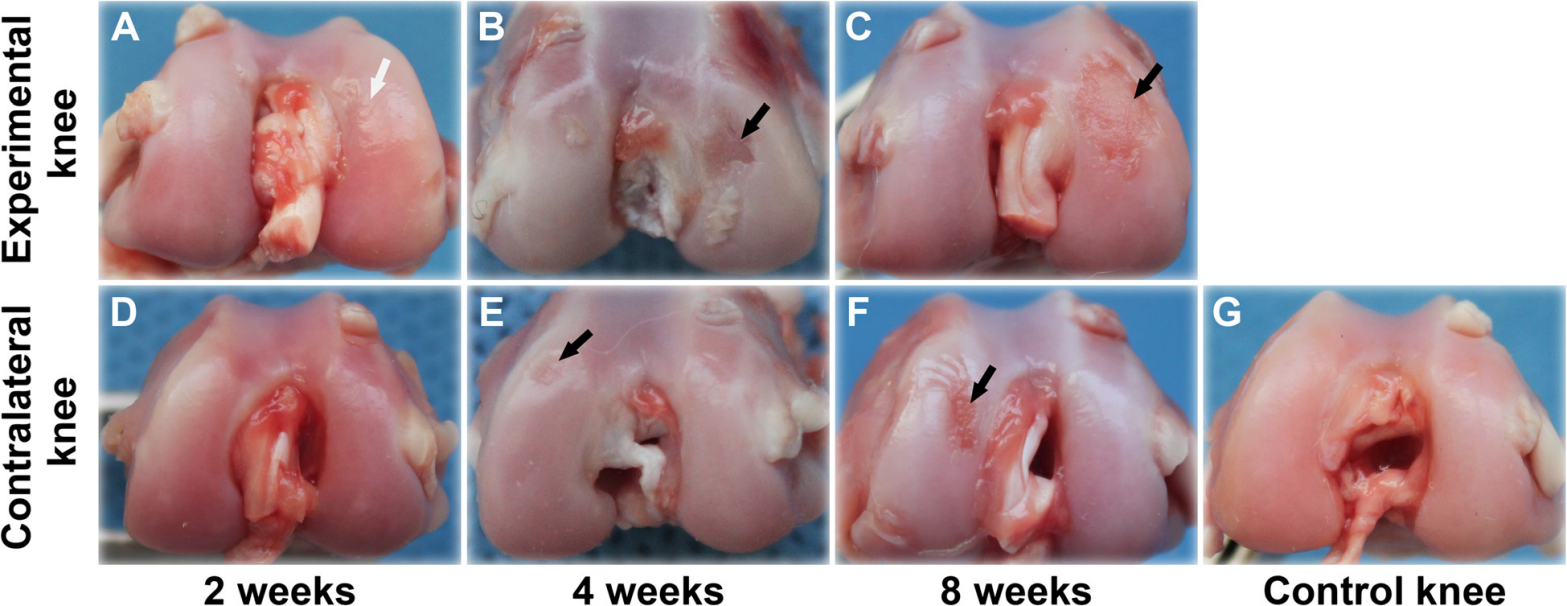
Macroscopic observation of cartilage specimens after immobilization. **a-c** Experimental knees; **d-f** Contralateral knees; **g** Control knee. White arrows: roughness of the cartilage surface. Black arrows: cartilage defect

In the experimental knees, the cartilage surface of medial femoral condyles showed roughness after 2 weeks of immobilization (Fig. 1a). Cartilage defects were detected after 4 weeks of immobilization (Fig. 1b), and defects were further developed after 8 weeks of immobilization (Fig. 1c). After 2 weeks of immobilization, cartilage surfaces of contralateral knees were smooth and shiny (Fig. 1d), similar to those of control knees (Fig. 1g). Cartilage surfaces of contralateral knees showed degenerative changes in the medial femoral condyles after 4 weeks of immobilization (Fig. 1e), and the degeneration showed further progression after 8 weeks of immobilization (Fig. 1f).

### Histological and immunohistochemical evaluation

**Fig. 2:**
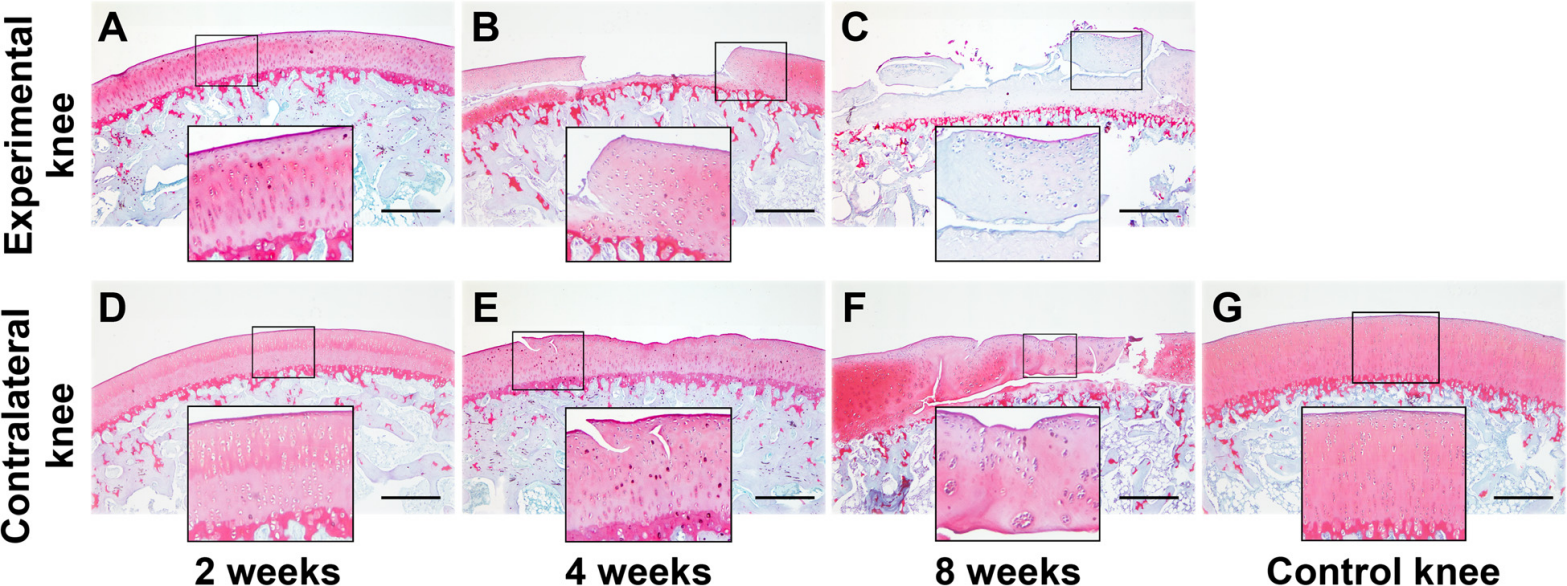
Safranin-O/Fast-Green staining of osteochondral specimens from the intermediate region of the medial femoral condyle after immobilization. **a-c** Experimental knees; **d-f** Contralateral knees; **g** Control knee. 40× magnification

After 2 weeks of immobilization, both the experimental and contralateral knees demonstrated smooth cartilage surfaces and normal cellular distribution (Fig. 2a, d), which resembled the control knees (Fig. 2g). However, SO staining was slightly reduced in the superficial cartilage layer in the experimental knees (Fig. 2a). After 4 weeks of immobilization, cartilage defects were detected, and hypertrophic chondrocytes and reduced SO staining were visible. Both were unevenly distributed in the cartilage of experimental knees (Fig. 2b). An irregular cartilage surface and cracks were found in the contralateral knees (Fig. 2e). After 8 weeks of immobilization, chondrocytes were partly lost or arranged in clusters, and SO staining was lost in the cartilage layer in the experimental knees (Fig. 2c). Hypertrophic chondrocytes and SO staining were unevenly distributed in the cartilage zone in the contralateral knees (Fig. 2f).

**Fig. 3:**
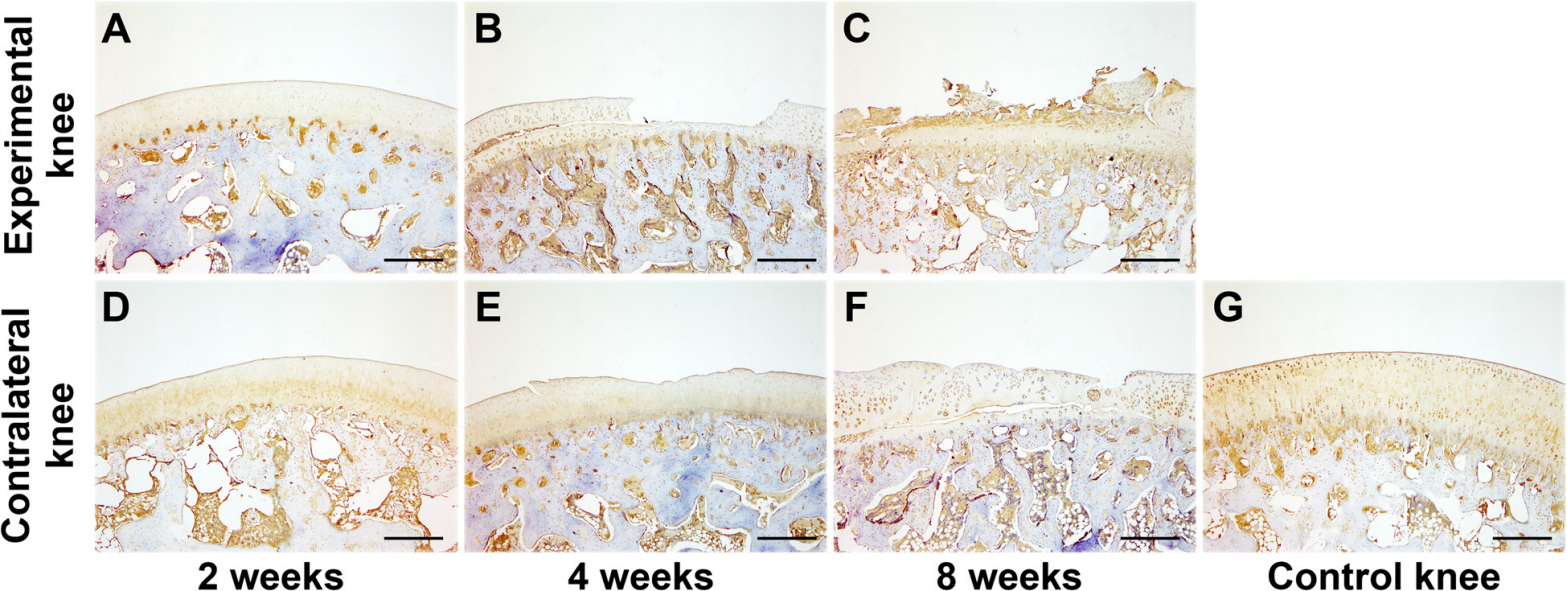
Type II collagen staining of osteochondral specimens from the intermediate region of the medial femoral condyle after immobilization. **a-c** Experimental knees; **d-f** Contralateral knees; **g** Control knee. 40× magnification

**Fig. 4:**
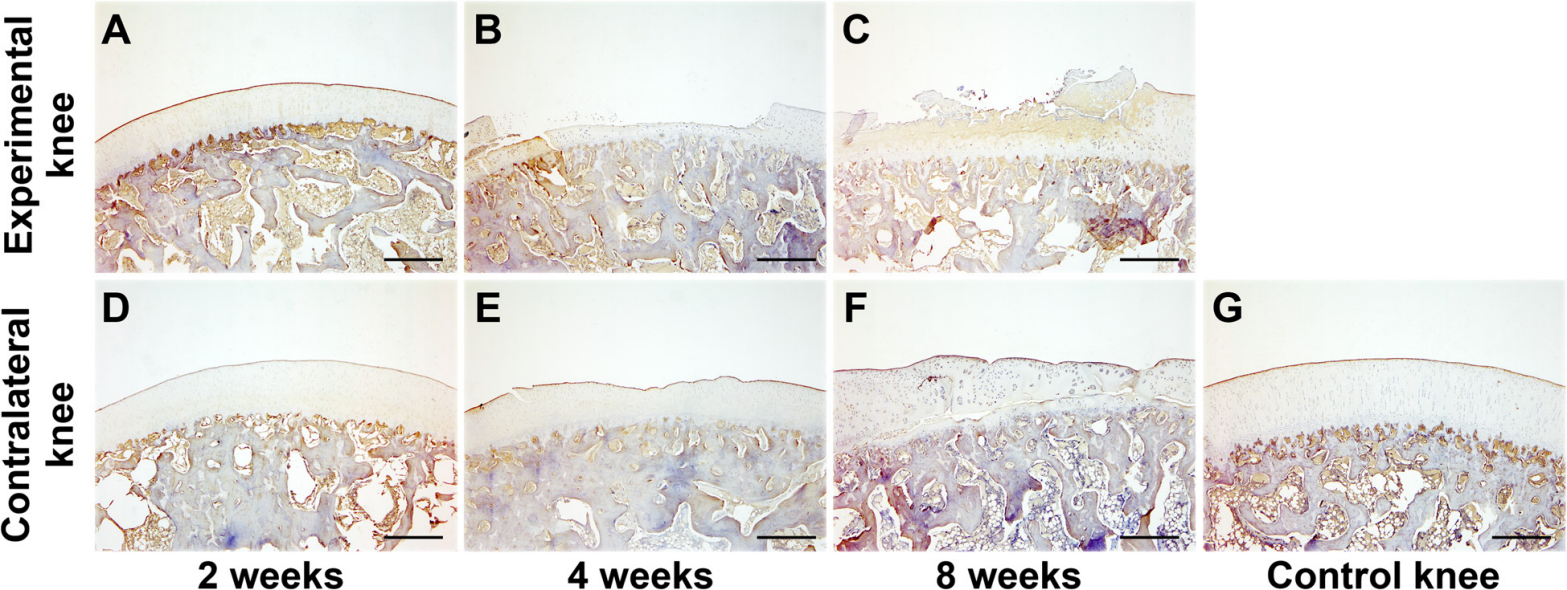
Type I collagen staining of osteochondral specimens from the intermediate region of the medial femoral condyle after immobilization. **a-c** Experimental knees; **d-f** Contralateral knees; **g** Control knee. 40× magnification

**Fig. 5:**
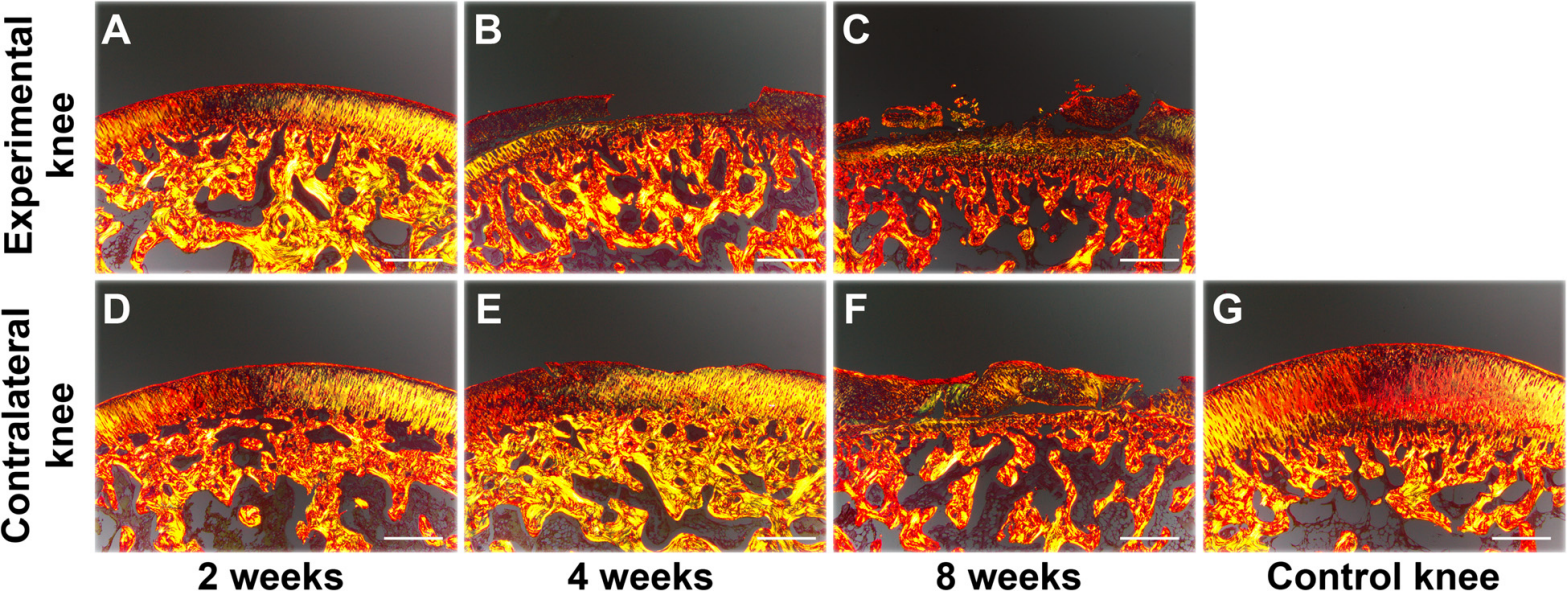
Sirius Red staining for cartilage anisotropy of osteochondral specimens from the intermediate region of the medial femoral condyle after immobilization. **a-c** Experimental knees; **d-f** Contralateral knees; **g** Control knee. 40× magnification

The pattern of type II collagen staining resembled that of SO staining. In the experimental knees, type II collagen staining was gradually reduced following immobilization (Fig. 3a-c). In the contralateral knees, type II collagen staining showed no obvious changes after 2 and 4 weeks of immobilization (Fig. 3d, e), when compared to the control knees (Fig. 3g). However, the staining of type II collagen was reduced after 8 weeks (Fig. 3f). For type I collagen distribution, no obvious changes were detected in the experimental knees after 2 and 4 weeks of immobilization (Fig. 4a, b) and in the contralateral knees after different periods of immobilization (Fig. 4d-f) when compared to the control knees (Fig. 4g). However, accumulation of type I collagen was obviously detected in the superficial and intermediate layers of cartilage in the experimental knees at 8 weeks (Fig. 4c). The collagen fiber orientation demonstrated no changes in both knees after 2 weeks of immobilization (Fig. 5a, d) when compared to the control knees (Fig. 5g). In addition, disruption of birefringence was gradually accelerated in both knees after 4 and 8 weeks of immobilization (Fig. 5b, c, e, F).

### Biochemical analysis

**Fig. 6:**
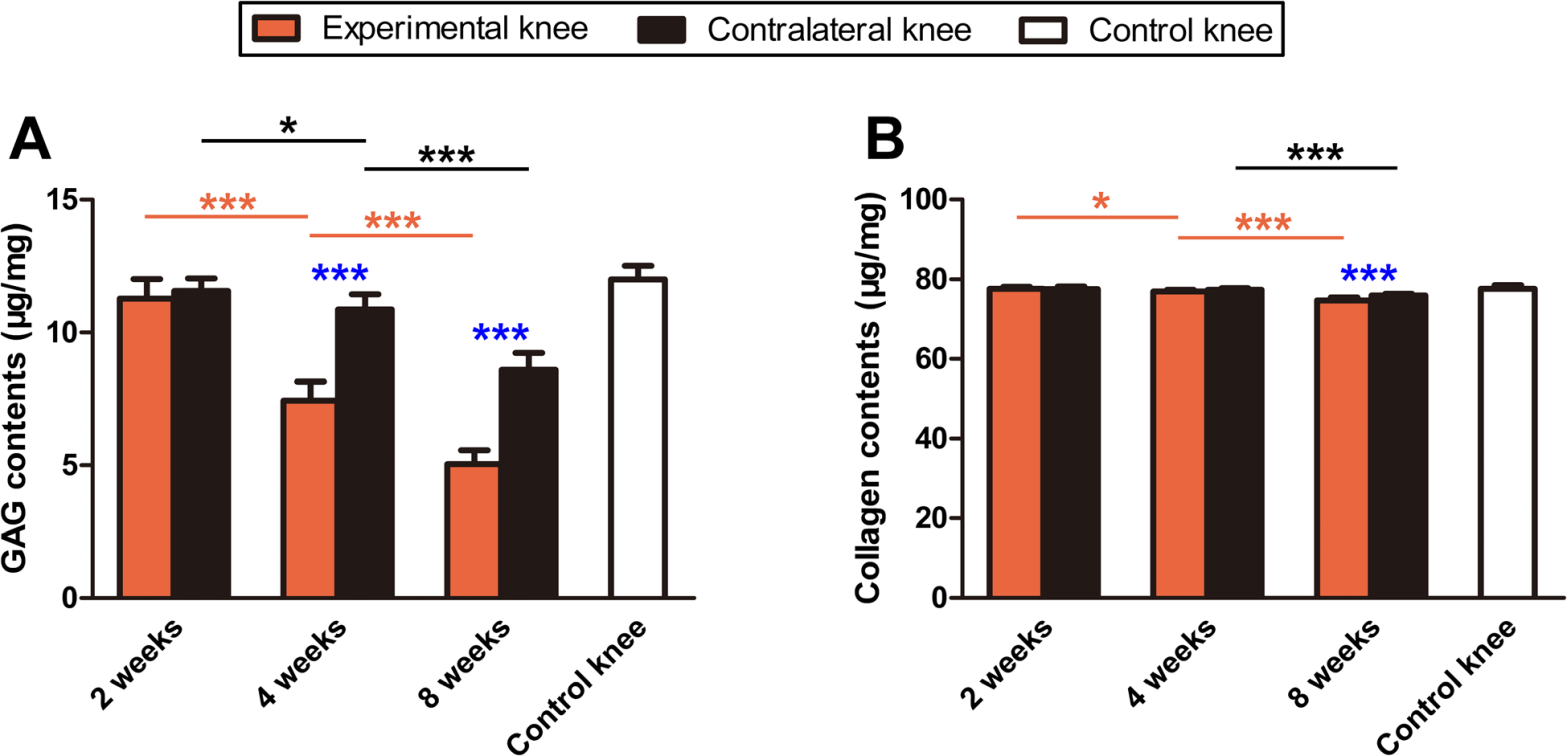
GAG and collagen content of cartilage specimens from the experimental and contralateral knees after immobilization. **a** GAG content; **b** Collagen content. *P < 0.05, ***P < 0.001. NS: no significant difference

GAG contents decreased gradually in both experimental and contralateral knees over time. The most significant decrease was observed in the experimental knees at 4 and 8 weeks and in the contralateral knees at 8 weeks. Moreover, the GAG content in experimental knees was significantly lower than in contralateral knees at 4 and 8 weeks (*P* < 0.001); however, no significant difference in GAG content between these two groups was detected at 2 weeks (*P* = 0.328; Fig. 6a).

The total collagen content of the experimental knees at 4 weeks was significantly lower than that at 2 weeks (*P* = 0.034); however, no significant difference between 2 and 4 weeks was detected in the contralateral knee (*P* = 0.513). The greatest decrease in collagen content was observed in both experimental and contralateral knees at 8 weeks. Furthermore, the collagen content in experimental knees was significantly lower than that in contralateral knees at 8 weeks (*P* < 0.001; Fig. 6b).

### Histopathological scores

**Fig. 7:**
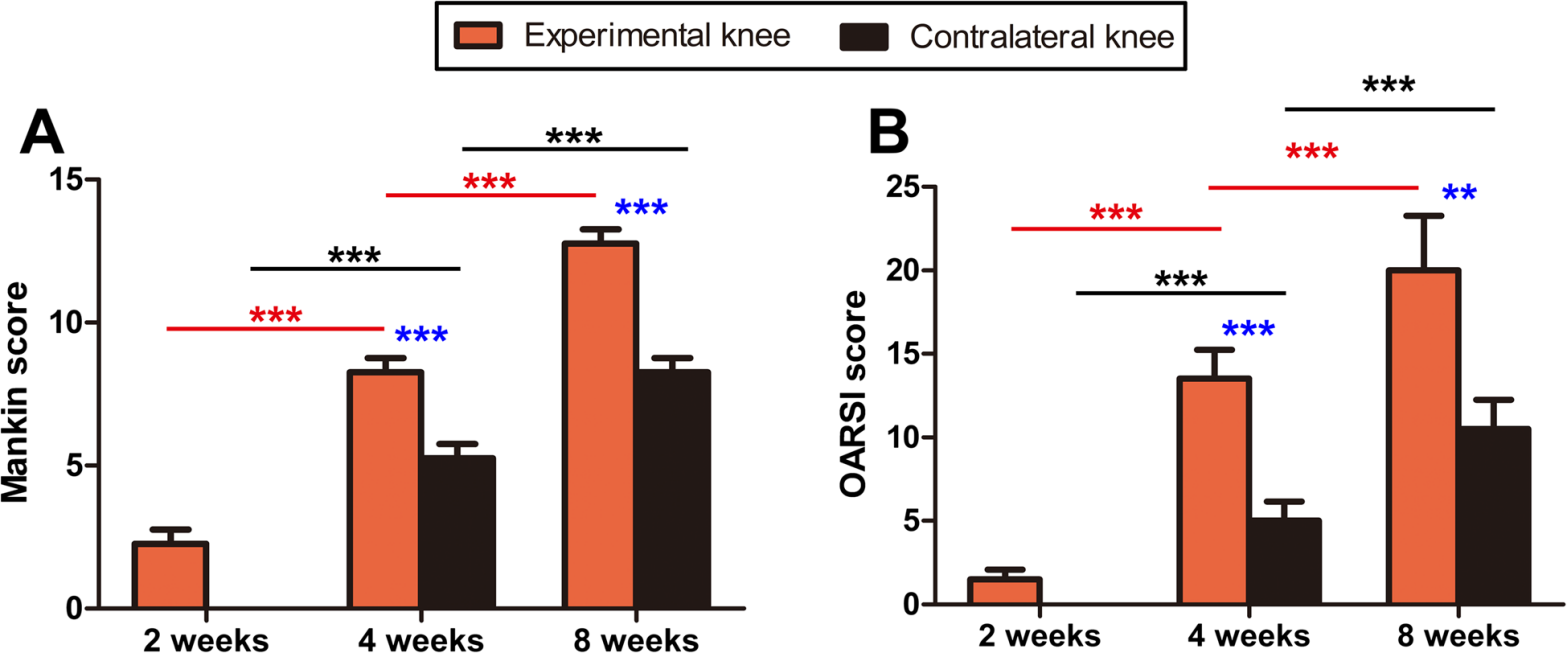
Mankin and OARSI scores of cartilage specimens from experimental and contralateral knees after immobilization. **a** Mankin score; **b** OARSI score. **P < 0.01, ***P < 0.001

Both Mankin and OARSI scores significantly increased in both experimental and contralateral knees with immobilization (*P* < 0.001; Fig. 7). Moreover, the Mankin score of experimental knees was significantly higher than that of contralateral knees after 4 and 8 weeks of immobilization, respectively (*P* < 0.001; Fig. 7a). The OARSI score of experimental knees was significantly higher than that of contralateral knees after 4 and 8 weeks of immobilization (*P* < 0.001 and *P* = 0.002; Fig. 7b).

## Discussion

In the present study, we determined the effect of unilateral immobilization of one knee on changes in the cartilage matrix of the contralateral knee in a rabbit model of OA. Our results demonstrate that OA developed in mobile knees following cartilage degeneration in the immobilized knees. To date, many groups have investigated the influence of joint immobilization on articular cartilage properties [10]. Normal articular cartilage has a smooth surface which appears white and shiny [18]. Jurvelin *et al*. immobilized the canine knee with a splint and detected the influence of immobilization on the thickness and stiffness of articular cartilage. They found that the thickness of cartilage was reduced and softening of cartilage surface developed after 11 weeks of immobilization [19]. Leroux and associates immobilized the canine knee with a cast for 4 weeks and investigated the changes in the mechanical behavior of tibial cartilage following immobilization. Their results showed that the mechanical properties of cartilage in both immobilized and contralateral knees were altered after 4-week immobilization when compared to the control knees collected from the non-immobilized canines [12]. In the present study, roughness of articular cartilage was observed visually in the immobilized knee (experimental knee) after 2 weeks of immobilization, and cartilage degeneration developed following immobilization. In the contralateral knees, cartilage surfaces demonstrated degenerative changes after 4 weeks, suggesting that immobilization caused joint surface degeneration in both immobilized and contralateral mobile knees.

In normal articular cartilage, the extracelluar matrix mainly consists of a network of collagen type II fibrils and proteoglycans, and collagens and proteoglycans are distinctively distributed in different anatomical zones of cartilage [20]. Behrens and coworkers immobilized dogs with casts or external fixators for 6 weeks and examined the effect of immobilization on the cartilage matrix. Their results showed that the proteoglycan content decreased in all joint locations in both casted and external fixator knees [21]. Haapala *et al.* . reported that GAG distribution of articular cartilage was changed and the largest reduction of GAG was detected in the superficial layer after 11 weeks of joint immobilization [11]. Moreover, Leroux *et al*. found that proteoglycan staining was significantly reduced in the cartilage after 4 weeks of immobilization; however, no significant influence of immobilization on the GAG content was detected following immobilization [12]. In our current study, SO staining and GAG content were gradually reduced in the experimental knees following immobilization. In addition, reduced SO staining and GAG content were detected in the contralateral mobile knee after 4 or 8 weeks of immobilization, indicating cartilage degeneration developed in the contralateral mobile knees following progression of OA in immobilized knees.

Furthermore, many groups reported that collagen distribution and content of articular cartilage demonstrated no significant changes following immobilization; however, the proportion of collagen cross-links was significantly reduced after a period of immobilization [11, 22, 23]. Our present study shows that type II collagen was gradually reduced following immobilization. Type I collagen accumulation was observed in the upper and middle layers of cartilage in the experimental knees at 8 weeks. Collagen orientation became gradually disorganized in both knees at 4 and 8 weeks of immobilization. In addition, significant reduction of collagen type II content was detected in both experimental and contralateral knees after an 8-week immobilization. The reduced integrity of the collagen fibril network influences the mechanical properties of articular cartilage [10], which might further cause changes in collagen content. In line with the results of macroscopic, histological, immunohistochemical, and biochemical evaluation, both Mankin and OARSI scores indicated that OA developed in the contralateral mobile knees in association with OA progression in the immobilized knees.

One of the putative reasons of OA development in the contralateral knees is that more focal mechanical loadings is applied to the contralateral mobile knees following OA progression in the immobilized knees [4, 24]. Furthermore, researchers reported that muscle mass and strength decreased with aging [25], and overload induced an increase in matrix metalloproteinases-2 activity which caused skeletal muscle hypertrophy [26]. Thus, such changes in the periarticular soft tissues such as muscle may play a role in the onset and development of OA in contralateral knees [25, 26].

There are some limitations of this study. Firstly, many researches reported that joint remobilization can prevent OA progression and restore the properties of articular cartilage in immobilized knees [11, 13, 27]. The effect of remobilization on occurrence and progression of OA in the non-immobilized knees will be further investigated in the future. Moreover, the present study investigated the effect of immobilization on cartilage matrix changes of the contralateral mobile knee in an immobilization-induced OA model. Whether other OA models (Anterior cruciate ligament transaction, meniscectomy, intraarticular injection, etc.) [28] can cause development of OA in the non-operated knee needs further investigation. Additionally, this study only investigated changes in the cartilage matrix of the medial femoral condyle (weight-bearing area). Further studies are needed to investigate changes in other sites, such as the trochlear groove, tibial plateau, menisci, synovium and subchondral bone.

## Conclusion

The present study demonstrated that OA developed in the contralateral mobile knees in association with the progression of OA in immobilized knees in a rabbit model of immobilization-induced OA.

## Abbreviations

OA: Osteoarthritis
TKA: Total knee arthroplasty
GAG: Glycosaminoglycan
SO: Safranin-O/Fast-Green
DAB: 3,3′-diaminobenzidine tetrahydrochloride
OARSI: Osteoarthritis Research Society International
SD: Standard deviation
